# Supplementary material for: The Changing Landscape of Food Deserts and Swamps over More than a Decade in Flanders, Belgium
Source: Int J Environ Res Public Health. 2022 Oct 25;19(21):13854. doi: 10.3390/ijerph192113854 (PMC9656286; doi:10.3390/ijerph192113854)
Supplement: Supplementary file 1 [file ijerph-19-13854-s001.zip › ijerph-1945535-supplementary.pdf]

Locatus Category	Explanation	Likert Scale Score	Healthy/unhealthy
**Primary food retailers**			
59.210.171-Fastfood	Meal provider where service is not at the table, without fixed cutlery and where the usually fried products are ready for consumption within minutes of ordering (excludes sandwich stores).	1	Unhealthy
59.210.215-Grillroom/Shoarma	Sales of grill products, shoarma, kebab and alike.	1	Unhealthy
59.210.180-Delivery	Provision of (hot) meals, which are not consumed on site but are collected or delivered.	2	Unhealthy
59.210.235-Hotel-Restaurant	Hotel combined with 'a la carte' restaurant.	3	Neutral
59.210.333-Lunchroom	Meal provider, with table service, particularly including breakfasts, lunches and desserts, opposed to restaurants mostly closed in the evening.	3	Neutral
59.210.430-Café-Restaurant	Provision of both beverages and meals.	2	Unhealthy
59.210.434-Restaurant	Provision of meals, beverages are provided only in conjunction with the food.	3	Neutral
59.210.392-Pancakes	Restaurants specializing in pancakes.	2	Unhealthy

11.010.519-Supermarkt	Stores with a wide and varied range of food products often supplemented by a narrow and shallow range of non-food products. Floor space is greater than 149m².	3	Neutral
11.010.309-Minisuper	See definition of supermarket, but store is up to 149m².	3	Neutral
11.010.012-Greengrocer	Almost exclusively sales of potatoes, vegetables and fruit that have not been cultivated by the company itself.	5	Healthy
11.010.471-Butcher	Sales of meat and meat products.	2	Unhealthy
11.010.399-Poulterer	Sales of game and poultry.	3	Neutral
11.010.588-Fish	Sales of fish, crustaceans and molluscs.	4	Healthy
11.010.261-Cheese	Sales of Cheese.	3	Neutral
11.010.111-Bakery	Sales of bread and pastries, with possible lunchroom but that is not be the main activity.	3	Neutral
11.010.112-Flans	Sales of pastries, with an emphasis on flans.	2	Unhealthy
11.010.310-On Farm Store	Farm sales of own products, primarily food products, supplemented by an assortment of purchased products.	4	Healthy
11.010.378-Nuts	Sales of nuts, dates and dried fruits.	4	Healthy

11.010.423-Bio Store	Biodynamic, ecological and macrobiotically grown products supplemented with dietary supplements, homeopathic remedies, herbs.	4	Healthy
11.010.132-Chocolate	Sales of Chocolate.	1	Unhealthy
11.010.657-Candystore	Sales of sweets and confectionery, also sugars.	1	Unhealthy
59.210.246-Icecream Parlor	Sales of ice cream.	1	Unhealthy
11.010.350-Nightshop	Mini supermarket with (late) evening and night opening.	2	Unhealthy
**Secondary food retailers**			Unhealthy
11.010.522-Tobacco	Sale of tobacco products and smoking accessories, in conjunction with magazines, confectionery and soft drinks.	1	Unhealthy
11.020.156-Drugstore	Sells personal care products, self-help medications, health care products, nursing and cleaning supplies.	2	Unhealthy
22.030.618-Warehouse	Store with a broad assortment of alimentary and non-alimentary products in which the fashion segment is often predominant.	2	Unhealthy
45.205.528-Gas station	Sale of automotive fuels, with or without a store.	2	Unhealthy

59.210.150-Disco	Opportunity for evening-night catering with a central dance floor and loud music.	1	Unhealthy
59.210.155 Sex/partyclubs	Opportunity for sex, erotic massages, shows, couples clubs etc.	1	Unhealthy
59.210.465-Partycenter	Rental of rooms for parties and celebrations, including the provision of food and beverages for these parties.	2	Unhealthy
59.230.018-Amusement hall	Is an age-independent, freely accessible place with gambling and gaming machines.	1	Unhealthy
59.230.020-Theme park	Terrain on which one or more attractions are assembled.	2	Unhealthy
59.230.028-Casino	An adult-only venue where you can play slot machines and various gambling games such as roulette and blackjack at a table in luxurious surroundings.	1	Unhealthy
59.230.078-Biljart/Pool	Space primarily designed for billiards/pool and / or snooker (any catering service here is additional and not the main purpose).	2	Unhealthy
59.230.080-Indoor playground	Indoor play area for children.	2	Unhealthy
59.230.102-Bowling	Bowling center (catering service is complementary here and not the main purpose).	2	Unhealthy
59.230.150-Zoo	Grounds where animals can be viewed for a fee.	2	Unhealthy

59.230.950-Amusement Other	All other forms of entertainment, not previously mentioned.	2	Unhealthy
65.250.033-Video Store	Rental of image and sound carriers with or without computer games.	1	Unhealthy
59.220.081-Cinema	Screening of films.	1	Unhealthy
59.220.549-Theater	Room for the screening of live shows.	2	Unhealthy
59.230.265-Go Cart Track	Place where karts can be rented and driven .	2	Unhealthy
59.230.295-Ice Skating Track	Permanently established indoor ice rink where artificial ice can be skated for a fee.	2	Unhealthy
59.230.290-Climbing Hall	Indoor facility for the practice of climbing .	2	Unhealthy
59.230.310-Laser Game	Center where laser games can be played.	2	Unhealthy
59.230.590-Ski Track	Indoor place where skiing can be done.	2	Unhealthy
59.230.700-Swimming Pool	Indoor public pool.	2	Unhealthy
59.230.570-Sauna	Sauna complex accessible to everyone.	2	Unhealthy
